# COVID-19 pandemic crisis—a complete outline of SARS-CoV-2

**DOI:** 10.1186/s43094-020-00133-y

**Published:** 2020-11-17

**Authors:** Sana Saffiruddin Shaikh, Anooja P. Jose, Disha Anil Nerkar, Midhuna Vijaykumar KV, Saquib Khaleel Shaikh

**Affiliations:** 1 Y. B. Chavan College of Pharmacy, Dr. Rafiq Zakaria Campus, Aurangabad, 431001 India; 2 Government College of Pharmacy, Aurangabad, 431001 India

**Keywords:** COVID-19, SARS-CoV-2, Lifecycle, Pathogenesis, Prevention, Clinical trials

## Abstract

**Background:**

Coronavirus (SARS-CoV-2), the cause of COVID-19, a fatal disease emerged from Wuhan, a large city in the Chinese province of Hubei in December 2019.

**Main body of abstract:**

The World Health Organization declared COVID-19 as a pandemic due to its spread to other countries inside and outside Asia. Initial confirmation of the pandemic shows patient exposure to the Huanan seafood market. Bats might be a significant host for the spread of coronaviruses via an unknown intermediate host. The human-to-human transfer has become a significant concern due to one of the significant reasons that is asymptomatic carriers or silent spreaders. No data is obtained regarding prophylactic treatment for COVID-19, although many clinical trials are underway.

**Conclusion:**

The most effective weapon is prevention and precaution to avoid the spread of the pandemic. In this current review, we outline pathogenesis, diagnosis, treatment, ongoing clinical trials, prevention, and precautions. We have also highlighted the impact of pandemic worldwide and challenges that can help to overcome the fatal disease in the future.

## Background

Coronaviruses (CoVs) are a large family of RNA viruses; they show discrete point-like projections over their surface. They show the presence of an unusually large RNA genome and a distinctive replication strategy. The term “coronavirus” is acquired from the “crown”-like morphology. Coronaviruses show potential fatal human respiratory infections and cause a variety of diseases in animals and birds [1]. Coronavirus primarily targets the human respiratory system [2]. The World Health Organization (WHO) named the latest virus as severe acute respiratory syndrome coronavirus 2 (SARS-CoV-2) on 12 January 2020 [3]. The COVID-19 or the SARS-CoV-2 is rapidly unfurling from Wuhan in Hubei Province of China to worldwide [4].

Initial confirmation of the pandemic was carried out by conducting studies on 99 patients with COVID-19 pneumonia, from which 49% of patients exhibited a history of subjection to the Huanan seafood market. The patient examined had a clinical manifestation of fever, cough, shortness of breath, muscle ache, and sore throat-like symptoms [5]. COVID-19 has infected several hundreds of humans and has caused many fatal cases [6]. Worldwide, there have been 3,925,815 confirmed cases, including 274,488 deaths of COVID-19 as of 6:37 pm CEST 10 May 2020 reported to WHO [7].

This article outlines and gives a complete overview of SARS-CoV-2, including its pathogenesis, diagnosis, treatment, prevention, and precautions. This article also provides the current scenario of the pandemic worldwide, since new findings are rapidly evolving and can help the readers in upgrading their knowledge about the COVID-19. It also emphasizes the challenges faced by giving an idea about future strategies in fighting and preventing recurrence.

## Main text

### History and origin

Coronaviruses were not expected to be highly infectious to humans, but the outburst of a severe acute respiratory syndrome (SARS) in Guangdong province China in the years 2002 and 2003 proved to be devastating. SARS-CoV is the contributory agent of the SARS, also known as “atypical pneumonia”. The coronaviruses that spread before that time in humans mostly caused mild infections in immune-competent people. But after the emergence of SARS, another highly infectious coronavirus, MERS-CoV, appeared in Middle Eastern countries [8, 9]. Research has shown that SARS-CoV-2 shows similarities with SARS-CoV and MERS-CoV. (Table 1) depicts a comparison of SARS-CoV-2 with SARS-CoV and MERS-CoV [10–16]. Several disseminating strains of coronaviruses were identified and were considered harmless pathogens, causing common cold and mild upper respiratory illness [17]. HCoV-229E [18] strain was isolated in 1966. HCoV-NL63 was first isolated from the Netherlands during late 2004. In 2012, MERS-CoV was first identified from the lung of a 60-year-old patient who was suffering from acute pneumonia and renal failure in Saudi Arabia [19]. About 8000 cases and 800 deaths worldwide were observed due to the outbreak of SARS first human pandemic in the dawn of the twenty-first century [20].

**Table 1:** Comparison of coronaviruses

Parameters	SARS-COV2	SARS-COV	MERS-COV
Epidemiology	Dec 2019, Wuhan, China	Nov 2002, Guangdong, China	April 2012, Saudi Arabia
Animal reservoir	Bats	Bats	Bats
Intermediate host	Pangolins/minks (yet to be confirmed)	Palm civets	Camels
Receptor target	ACE2	ACE2	DPP4
Fatality rate	2.3%	9.5%	34.4%
Genetic similarity with the other	79.5% SARS-CoV 50% MERS-CoV	79.5% SARS-CoV-2	50% SARS-CoV-2
Virus type	SS-RNA	RNA	RNA
Total RNA sequence length of pathogen	29,903 bp	29,751 bp	30,108 bp
M:F ratio	2.70:1	1:1.25	2:1
Transmission route	Droplets; faeco-oral transmission; contact with infected individual or things Human-to-human	Droplets; contact with infected individual or things; bat-civets-human Human-to-human	Touching infected camel or consumption of meat or milk Limited human-to-human transmission
Clinical symptoms	Fever, fatigue, dry cough	Fever, cough, myalgia, dyspnea, diarrhea	Fever, cough, respiratory distress, vomiting, diarrhea
Incubation	7–14 days, 24 days	2–7 days	5–6 days
R_0_	2.68	2.5	> 1
Diagnostic methods	RRT-PCR, RT-PCR, RT-lamp, RRT-lamp, coronavirus detection kit	RRT-PCR, RT-PCR, RT-lamp, RRT-lamp, coronavirus detection kit	RRT-PCR, ELISA, micro neutralization assay, MERS-CoV serology test
Chest X-ray	Bilateral multi-lobular ground glass opacities	Ground glass opacities	Ground glass opacities; consolidation
Chest CT scan	No nodular opacities	Lobar consolidation; nodular opacities	Single or multiple opacities; bilateral glass opacities; sub-pleural and lower lobe predominance; septal thickness
Prevention	Hand hygiene; cough etiquette; avoiding unnecessary touching of the eyes or face.	Hand hygiene; cough etiquette; avoiding unnecessary touching of the eyes or face.	Hand hygiene; cough etiquette; avoiding unnecessary touching of the eyes or face; avoiding raw milk and meat consumption.
Treatment	Ritonavir; lopinavir (in testing)	Glucocorticoids; interferon	Ribavirin; interferon; analgesics (treatment not yet determined)

Note: despite the lower case fatality rate observed in COVID-19, the overall number of death far outweighs that from SARS and MERS

The α-CoVs HCoV-229E and HCoV-NL63 and β-CoVs HCoV-HKU1 and HCoV-OC43 are identified as a human susceptible virus with low pathogenicity and cause mild respiratory symptoms similar to common cold [21]. SARS-CoV and MERS-CoV result in severe respiratory tract infections [22, 23]. COVID-19 was recently reported from Wuhan (China), which has cases in Thailand, Japan, South Korea, and the USA, which has been confirmed as a new coronavirus [24].

The coronavirus genera, which mostly infect mammals, are alpha-coronavirus and beta-coronavirus. Out of 15 presently assigned viral species, seven were isolated from bats. The research proposed that bats are significant hosts for alpha-coronaviruses and beta-coronaviruses and play an essential role as the gene source in the evolution of these two coronavirus genera SARS and MERS [25]. The genome sequence was found to be 96.2% identical to a bat CoV RaTG13, whereas it shares 79.5% identity to SARS-CoV. The virus genome sequencing outcomes and evolutionary analysis show that bat can be a natural host from virus source, and SARS-CoV-2 might be transferred from bats through unspecified intermediate hosts to infect humans [26]. It is found that SARS-CoV-2 affects males more than females [27]. The spread of SARS-CoV-2 emerged like a wild forest fire in many countries worldwide. Table (2) [28] gives a brief of the first identified cases of COVID-19 in different countries.

**Table 2:** First confirmed case

Country	First confirmed case (dates)
China, East Asia	31 December 2019
Thailand	13 January 2020
Japan	15 January 2020
Korea	20 January 2020
USA	23 January 2020
Vietnam	24 January 2020
Singapore	24 January 2020
Australia, Nepal, and French Republic	25 January 2020
Malaysia	26 January 2020
Canada	27 January 2020
Cambodia, Germany, Sri Lanka	28 January 2020
United Arab Emirates	29 January 2020
Philippines, India, Finland	30 January 2020
Italy	31 January 2020
Russian Federation, Spain, Sweden, UK	1 February 2020
Belgium	5 February 2020
Japan	6 February 2020
Egypt	15 February 2020

The first confirmed case was reported in China, and since then, there was a widespread of coronavirus in other countries worldwide. Table 1 shows the first confirmed case with dates

### Structure

Coronaviruses are spherical to pleomorphic enveloped particles [29]. The size ranges from 80 to 120 nm in diameter. The maximum size is as small as 50 nm and as large as 200 nm are also seen [30]. There are four types of main structural proteins observed in the coronaviruses: the spike (S), membrane (M), envelope (E), and nucleocapsid (N) proteins, which are encoded within the viral genome (Table 3). In thin sections, the virion envelope may be visualized as inner and outer shells separated by a translucent space [31]. The virion envelope contains phospholipids, glycolipids, cholesterol, di- and triglycerides, and free fatty acids in proportions. The complexed genome RNA is with the basic nucleocapsid (N) protein, which forms a helical capsid established within the viral membrane. The enclosed glycoproteins are responsible for attachment to the host cells [32].

**Table 3:** Structural proteins of coronavirus and their functions

Structural proteins	Functions of proteins
Spike protein (S)	Virus and host cell fusion by binding
Membrane protein (M)	Nutrient transport, determines shape, and formation of envelope
Envelope protein (E)	Interferes with host immune response
Nucleocapsid protein (N)	Binds with RNA genome and makes up nucleocapsid
Hemagglutinin-esterase (HE)	Binds sialic acids on surface glycoprotein

According to the recent studies, it is observed that coronavirus which lacks envelope protein (E) serves as a good candidate in vaccine designing

**Fig. 1:**
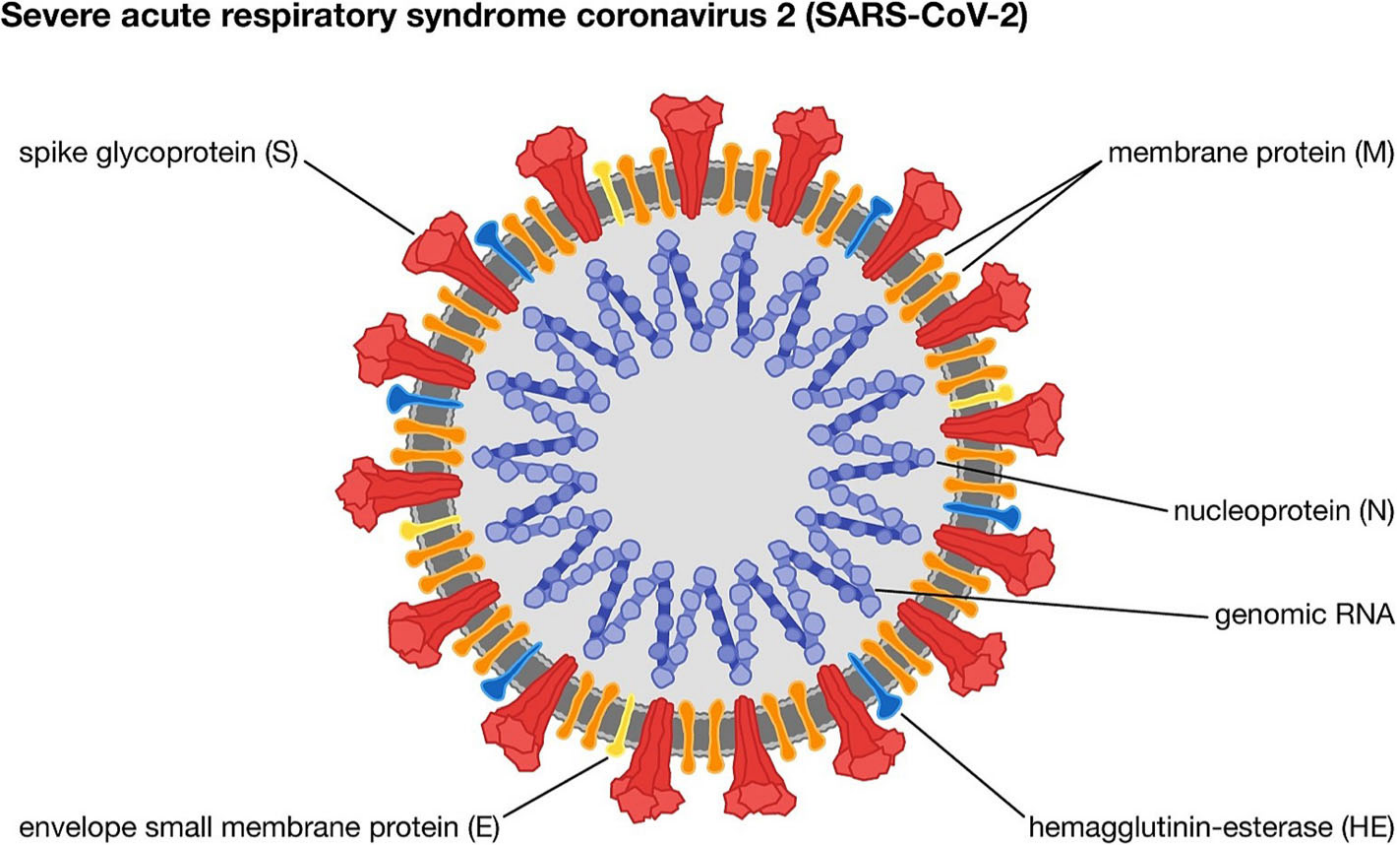
Structure of novel coronavirus

The coronavirus genomes are among the most massive mature RNA molecules as compared to other eukaryotic RNAs (Fig. 1) [33]. The genome of these viruses contains multiple ORFS. A typical CoV consists of at least 6 ORFs in its genome. Several studies have confirmed the genetic resemblance between SARS-CoV-2 and a bat CoV.

**Fig. 2:**
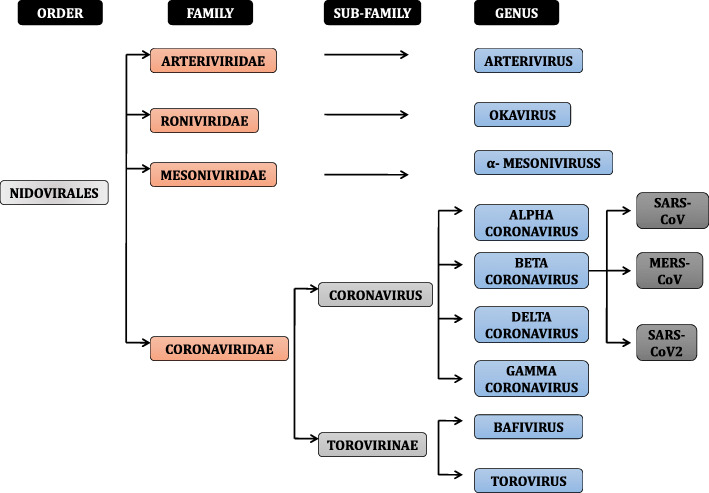
Classification of coronavirus

A study conducted to compare the genetic mutations of COVID showed genomic mutations among viruses from different countries, wherein a sequence obtained from Nepal showed minimum to no variations. In contrast, the maximum number of modifications was obtained from one derived from the Indian series located in ORF1-ab nsp2 nsp3 helicase ORF8 and spike surface glycoprotein. Also, host antiviral mRNAs play a critical part in the regulation of immune response to virus infection, depending upon the viral agent. The unique host mRNAs could be explored in the development of novel antiviral therapies. The club-like surface projections or peplomers of coronaviruses are about 17–20 nm from the virion surface. It has a subtle base that swells to a width of about 10 nm at the distal extremity. Some coronaviruses that exhibit the second set of projections about 5–10-nm long are present beneath the significant projections. These shorter spikes are now known as hemagglutinin-esterase (HE) protein, an additional membrane protein found in a subset of group 2 coronaviruses. The primary role of this non-essential protein is to aid in viral entry and pathogenesis in vivo. It configures short projections that bind to N-acetyl-9-O-acetlyneuramic acid or N-glycolylneuraminic acid and have esterase [34–39]. Figure 2 shows the primary classification of coronavirus.

### Lifecycle of coronavirus

**Fig. 3:**
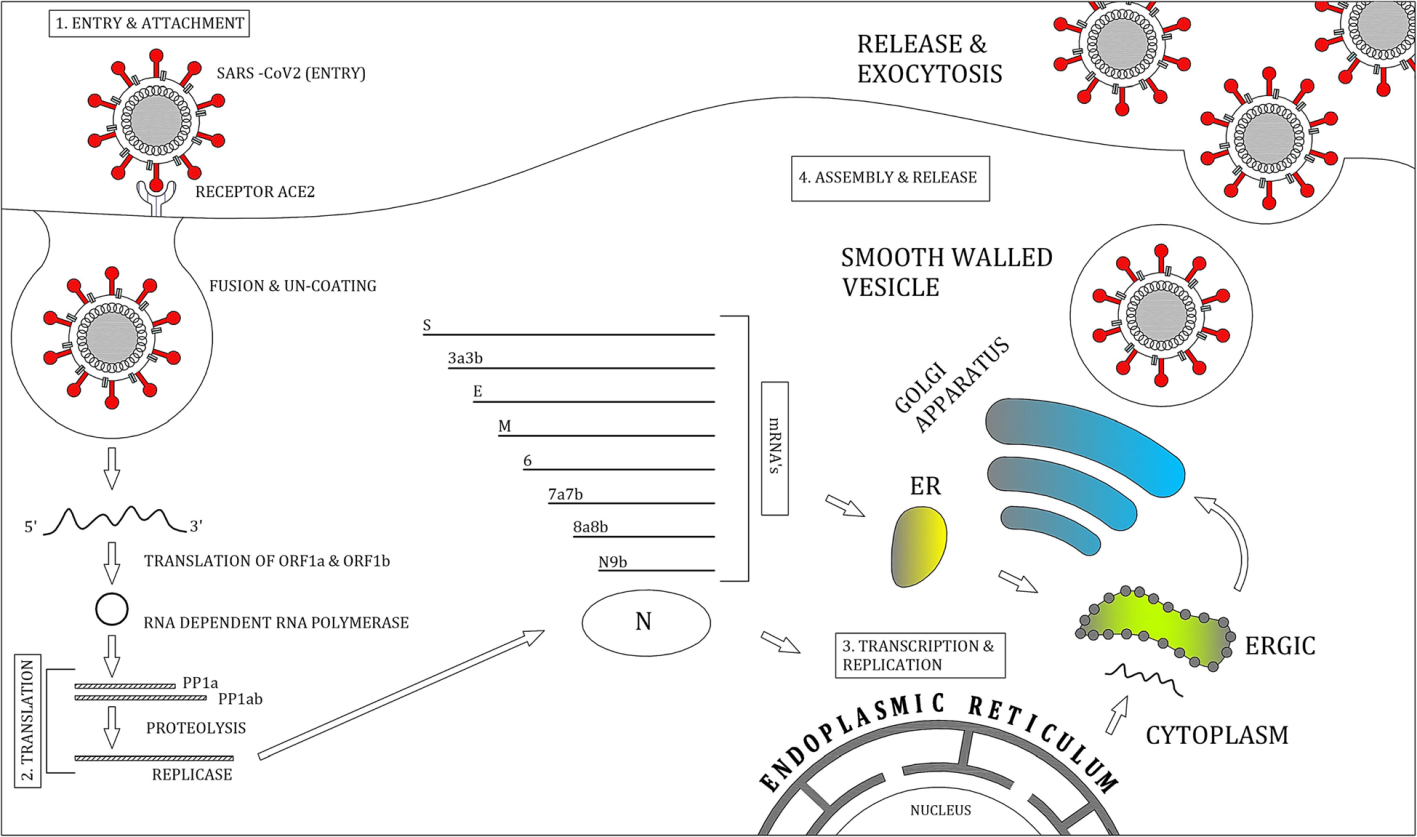
Lifecycle of coronavirus

The life cycle of the virus with the host consists of the following four steps: attachment, penetration, biosynthesis, maturation, and release (Fig. 3). Once the virus binds to the host receptor, they enter host cells through endocytosis or membrane fusion. Once the viral contents are released inside the host cells, viral RNA enters the nucleus for replication. Viral mRNA is used to make viral proteins and is further proceeded by maturation and release [40, 41].

#### Attachment and entry

The virion attachment with the host cell is initiated by interaction between S protein and its receptors, which is also a primary determinant for coronavirus infection. The S protein undergoes acid-dependent proteolytic cleavage, which results in exposure of fusion peptide. This fusion is followed by the formation of a six-helix bundle (bundle formation helps in combining viral and cellular membrane) and release of the viral genome into the cytoplasm.

#### Replicase protein expression

The process of translation of replicase gene ORFs1a and ORFs1b and translation of polyprotein pp1a and pp1ab takes place. Assembly of nsps into replicase-transcriptase complex (RTC) leads to viral RNA synthesis (replication and transcription of subgenomic RNAs).

#### Replication and transcription

In the replication process, the viral RNA synthesis is followed by the production of genomic and sub-genomic RNAs (sub-genomic mRNAs), which further leads to recombination of the virus.

#### Assembly and release

The insertion and translation of viral structure protein S, E, and M takes place into the endoplasmic reticulum (ER), which is followed by the movement of proteins along the secretory pathway into ERGIC (endoplasmic reticulum Golgi intermediate compartment). The viral genome is encapsidated by N protein into the membrane of ERGIC. M and E protein expression give rise to the formation of virus-like particles (VLPs). After the assembly of the virion and its transportation to cell surface vesicles, exocytosis takes place. Finally, it results in viral release (E protein helps by altering the host secretory pathway).

### Incubation

The incubation period is the period between the entry of the virus into the host and appearance of signs and symptoms in the host or the period between the earliest date of contact of the transmission source and the most initial time of symptom onset (i.e., cough, fever, fatigue, or myalgia) [42]. The incubation period of COVID-19 is vital as the disease could be transmitted during this phase through asymptomatic as well as symptomatic carriers (Table 4). The inhaled virus SARS-CoV-2 binds to the epithelial cells present in the nasal cavity and starts replicating.

**Table 4:** Incubation period of coronaviruses

Coronavirus strain	Incubation period	Death period	Symptoms
SARS-CoV	4–10 days	20–25 days	Fever, dry cough, myalgia, dyspnea, headache, sore throat, sputum production, rhinorrhea, watery diarrhea, confusion, poor appetite.
MERS-CoV	5–6 days	11–13 days	Myalgia, fever, chills, malaise associated with confusion, cough, shortness of breath, dyspnea, pneumonia
COVID-19	3–7 days	17–24 days	Fever, cough, dyspnea, muscle ache, confusion, headache, sore throat, rhinorrhea, chest pain, diarrhea, nausea, vomiting, anosmia, dysgeusia

On the basis of studies conducted and data findings, virologists points out that incubation period extends to 14 days, with a median time of 4–5 days from exposure to symptom onset. One study reported that 97.5% of persons with COVID-19 who develop symptoms will do so within 11.5 days of SARS-CoV-2 infection

ACE2 is the primary receptor for both SARS-CoV-2 and SARS-CoV, which is an asymptomatic state (initial 1–2 days of infection). Upper airway and conducting airway response are seen the next few days. The disease is mild and mostly restricted only to the upper conducting airways for about 80% of the infected patients [43].

The incubation period is required to create more productive quarantine systems for people infected with the virus. The incubation period for the COVID-19 is between 2 and 14 days after exposure. A newly infected person shows symptoms in the about 5 days after contact with a sick patient. In most patients, symptoms appeared after 12–14 days of infection

**Fig. 4:**
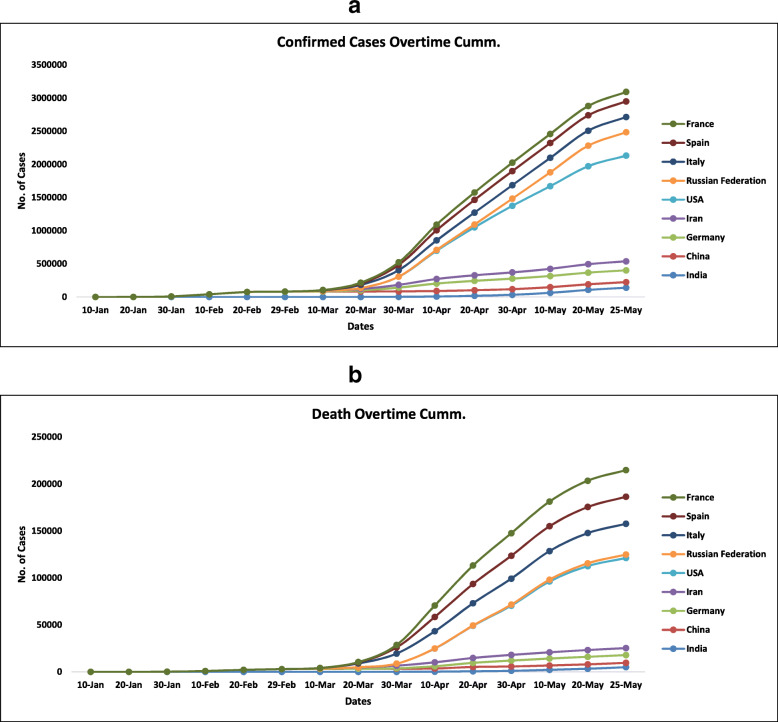
**a** Graph of confirmed (cumulative) cases overtime in various countries*.* **b** Graph of death (cumulative) overtime in various countries

The average incubation period was approximated to be 5.1 days, and 97.5% of those who develop symptoms will do so within 11.5 days of infection. In Wuhan’s return patients, the average incubation period is found to be 6.4 days. In a case reported by Hubei province, local government on 22 February showed an incubation period of 27 days. In another case, an incubation period of 19 days was observed. Therefore a 24-day observation period is considered in suspected cases by the Chinese government and also by WHO [44–51]. The frequency of cases is increasing day by day, and it is essential to keep a check over it. Figure 4 gives a glance of confirmed cases cumulative and death overtime cumulative from 10 January onwards up to 25 May.

### Pathogenesis

**Fig. 5:**
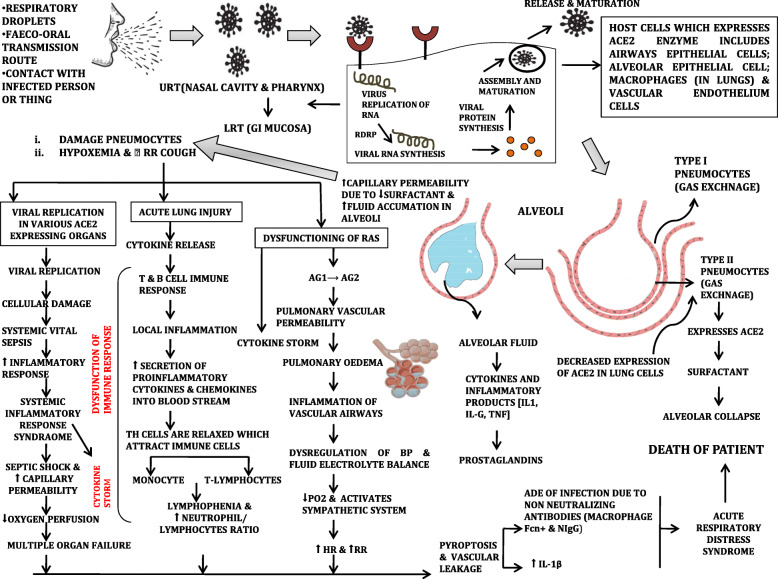
Complete pathogenesis of coronavirus

Like other CoVs, the SARS-CoV-2 is transmitted primarily via respiratory droplets and possible faeco-oral transmission routes [52]. Figure 5 gives a complete outline of the pathogenesis of coronavirus. On infection, primary viral replication is expected to occur in the mucosal epithelium of the upper respiratory tract with further multiplication into the lower respiratory tract and GI mucosa, giving rise to mild viremia. The virus enters the host cells through two methods either:

I. Direct entry
II. Endocytosis

These are positive sense ss-RNA viruses that can cause respiratory, enteric, hepatic, and neurologic diseases. High binding capacity with SARS-CoV-2 was observed by molecular biological analysis [53]. The ACE2 gene encodes the angiotensin-converting enzyme-2 receptor for both the SARS-CoV and the human respiratory coronavirus NL63. Recent studies show that ACE2 could be the host receptor for the novel coronavirus 2019-nCoV/SARS-CoV-2 [54].

Human angiotensin-converting enzyme 2 (hACE2), which was the binding receptor of SARS-CoV, is analogous to SARS-CoV-2. These hACE2 are type 1 membrane proteins expressed in various cells of the nasal mucosa, lung, bronchus, heart, kidney, intestines, bladder, stomach, esophagus, and ileum. It functions as an enzyme in the RAS and is, therefore, mainly associated with cardiovascular diseases [55].

The zinc peptidase ACE2 has also expressed in the alveolar type 2 pneumocytes, which explains its role in lung damage due to SARS-CoV. The SARS-CoV-2 shows 10–12-fold more affinity towards the proteins than the other SARS-CoV. Pathophysiology and virulence of the virus link to the function of its nsps and structural proteins. The nsp can block the host’s innate mechanism response while the virus envelope increases the pathogenicity as it assists the assembly and release of the virus [56].

The CoV spike glycoproteins comprise of three segments—a large ectodomain, a single-pass transmembrane anchor, and a small intracellular tail. The ectodomain is composed of the receptor-binding domain (RBD)—the S1 and the membrane fusion subunit S2. The two significant areas in s1, N-terminal domain (NTD) and the c-terminal domain (CTD), have been identified. The S1 NTDs are essential for binding to the sugar receptors, and the s1 CTDs are responsible for binding receptors ACE2, SPN, and DPP4 [57]. The S proteins undergo a considerable structural rearrangement to fuse with the viral membrane of the host cell membrane. The s1 subunit shedding and the s2 subunit transition to a highly stable conformation is the initial step in the fusion process [58]. The ACE2 consists of the N-terminal peptidase domain (NPD) and the C-terminal collectrin-like domain (CCTD) that ends with a single transmembrane helix and a 40 residue intracellular segment. It provides a direct binding site for S protein of CoVs.

The enzymes which assist this virus attachment include the serine protease enzymes TMPRSS2. These enzymes, which are cell-surface proteases, facilitate entry. In endosomes, the S1 of s proteins is cleaved, and the fusion peptides S2 are exposed. This exposed S2 unit brings the HR1 and HR2 together, resulting in membrane fusion and thereby release of viral package into the host membrane [59].

The viral RNA enters the nucleus for replication after the viral contents are released. Viral mRNA is used to make viral proteins. Decreased expression of ACE2 in a host cell results in an attack on the airway epithelium by the virus. These lead to acute lung injury that triggers immune responses. The release of various pro-inflammatory and chemokines like IL-6, IFN- gamma, MCPI 1, and IL-10 leads to capillary permeability in alveolar sacs. Due to local inflammation in the lungs, the secretion of pro-inflammatory cytokines and chemokines increases into the blood circulation of the patient. It results in fluid filling and increased difficulty in the exchange of gases across the membrane. Viral replication and infection in airway epithelial cells could cause high levels of virus-linked pyroptosis with associated vascular leakage. IL-beta cytokine released during pyroptosis is a highly inflammatory form of programmed cell death, which is the trigger subsequent inflammatory response. The IgG antibodies against SARS-CoV-2 N protein can be detected in the serum in the early stages at the onset of the disease. The non-neutralizing antibodies result in ADE (antibody-dependent enhancement), which leads to an increased systematic inflammatory response.

The pro-inflammatory cytokines and chemokines are an indicator of T_H_ cells. Secretions from such cytokines and chemokines attract immune cell monocytes and T lymphocytes. High levels of pro-inflammatory cytokines, including IL-2, IL-7, IL-10, IP-10, G-CSF, MCP-1, MIP-1A, and TNF alpha, were detected in the severe infection called cytokine storm or cytokine release syndrome as a crucial factor in the pathogenesis of COVID-19.

The cytokine storm increases the inflammatory response resulting in increased blood plasma levels of neutrophils IL-6, IL-10, granulocytes, MCP1, TNF, and decreased organ perfusion, which results in multiple organ failure. Cytokine storm and pulmonary edema due to ACE2 dysregulation result in acute respiratory distress syndrome. SARS-CoV-2 can also affect the CNS [60]. Myocardial damage increases the difficulty and complexity of patient treatment [61]. Clinical investigations have shown that patients with cardiac diseases, hypertension, or diabetes, who are treated with ACE2-increasing drugs, including inhibitors and blockers, are at higher risk of getting infected with SARS-CoV2 [62]. Death results due to ARDS and multiple organ failure.

### Symptoms

**Fig. 6:**
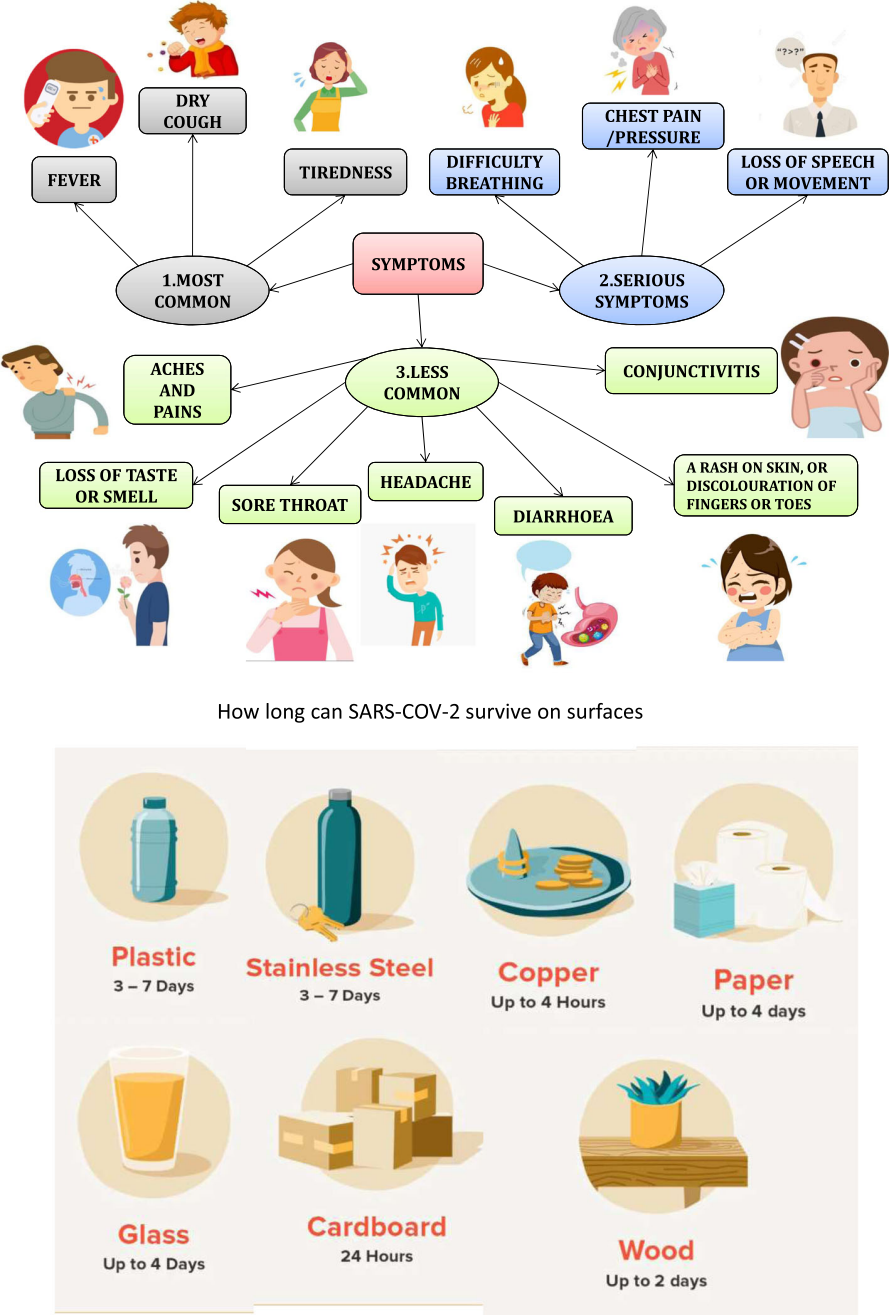
Symptoms for coronavirus

People with COVID-19 infection show symptoms ranging from mild to severe illness. Figure 6 shows a brief outline of various symptoms related to COVID-19. The warning signs and symptoms such as trouble breathing, constant pain or pressure in the chest, inability to wake or stay awake, and bluish lips or face are observed in patients [63]. Older people (65 years and older) are at higher risk of developing the disease.

**Fig. 7:**
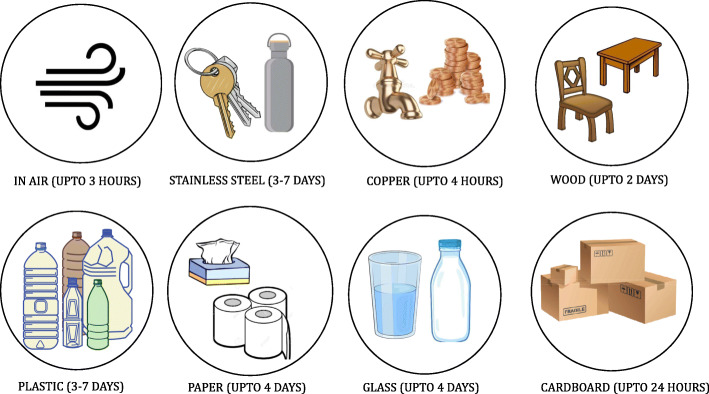
Survival of virus on various objects

According to a study, people of all ages having asthma, diabetes, HIV, liver diseases, severe heart conditions, severe obesity (body mass index [BMI] of 40 or higher), and chronic kidney diseases undergoing dialysis show a higher mortality rate. The other populations with people showing disabilities, pregnancy, and breastfeeding and people experiencing homelessness, racial, and minority groups are at elevated risk of transmission of disease [64]. The crucial fact to know about coronavirus on surfaces is that they can easily be cleaned with ordinary household disinfectants that will kill the virus [65]. Studies have shown (Fig. 7) that the COVID-19 virus can survive for up to 72 h on plastic and stainless steel, about 4 h on copper, and less than 24 h on cardboard [66].

### Diagnosis: COVID-19

There are two categories of tests available for COVID-19:

- Viral tests: a viral analysis indicates whether a person has a current infection.
- Antibody tests: an antibody indicates whether a person had an infection.

The protection of getting infected again in a person showing the presence of antibodies to the virus is still unexplained [67].

#### Tests for current infection

A swab sample is collected (from the nose) to conclude that a person is currently infected with SARS-CoV-2. Some tests are called as point-of-care tests, which means their results may be available in less than an hour. Other test takes 1–2 days for analyzing after being received by the laboratory [68].

#### Test for past infection

Antibody tests analyze a blood sample for the presence of antibodies, which show if one had a previous infection with the virus. Antibody tests cannot be used to diagnose someone as being currently infected with COVID-19. Antibody tests are accessible through healthcare providers and laboratories [69]. In severe cases, clinical diagnosis is done based on the clinical manifestations of respiratory failure syndrome, increased liver function tests, blood tests indicating leukopenia, and high levels of ferritin. For such, a test for soluble CD-163 (sCD-163), showing the activation of macrophages, was suggested [70]. Laboratory diagnosis included genomic sequencing, reverse-transcription polymerase chain reaction (RT-PCR), and serological methods (such as enzyme-linked immunoassay [ELISA]). Because of the rapidly changing diversity found in the expression of the novel coronavirus, pneumonia became diverse and quickly changed. Other methods used are radiographic images for early observations and evaluation of disease severity [71].

Reverse-transcription polymerase chain reaction (RT-PCR) shows high sensitivity for new SARS cases. The suspected cases must be confirmed by using RT-PCR and other methods (slower methods) of detection such as serology or viral culture, isolation, and identification by electron microscopy, thereby causing a significant increase in the time required for an accurate diagnosis [72]. The samples are collected from upper and lower respiratory tracts through expectorated sputum, bronchoalveolar lavage, or endotracheal aspirate, which are then assessed by conducting polymerase chain reaction for viral RNA. It is recommended to repeat the test for reevaluation purposes in case of a positive result, and if the test is negative, a strong clinical impression also permits repeat testing [73].

An alternative diagnostic test to detect the SARS-CoV is mass spectroscopic identification of microbial nucleic acid signatures. Computed tomography images of the lungs showed 100% multiple patchy with fine mesh and consolidated shade distributed under the pleura. Nucleic acid tests were conducted in 187 patients, and all were positive to SARS-CoV-2. In the pulmonary CT images, 8% of them (15 cases) showed diffused lesions in either lungs or white lung. In the absorptive period, 98.9% showed fibrogenesis and diminished lesions. The CT imaging features differed from each follow-up showing different clinical symptoms [74]. The improvement in the detection of COVID-19 was found by the ELISA method. It is based on SARS r-CoV Rp3 nucleocapsid protein, which helps to detect the IgM and IgG against SARS-CoV-2. ELISA is a highly recommended method as the sampling blood is less stringent, and antibodies allow longer windows than oropharyngeal swabs for detecting viruses [75].

### Treatment

There is no particular treatment recommended for COVID-19. There is no data obtained regarding prophylactic treatment for COVID-19, only we can prevent from coming in contact with the pathogen. Confirmed cases are hospitalized and admitted in the same ward. Patients with mild symptoms may not require hospitalization [76]. They are isolated or self-isolated at home by following the doctor’s advice. Critically ill patients (respiratory shock, respiratory failure, septic shock, or other organ failures) should be admitted to ICU as soon as possible [77].

#### General treatment

The general treatment includes bed rest and supportive measures ensuring sufficient intake of calories, fluid, and electrolytes, and maintenance of acid-base homeostasis. Monitoring oxygen saturation and vital signs, keeping the respiratory tract unobstructed and inhaling oxygen, measuring C-reactive protein, hematology and biochemistry laboratory testing and ECG, blood gas analysis, and examining of chest images as when required and monitoring for any complications [78]. Patients having high body temperature above 38.5°C Celsius are administered with ibuprofen and acetaminophen orally.

#### Oxygen therapy

Patients with conditions of obstructed breathing, respiratory distress, shock, coma, and convulsions must receive oxygen therapy and airway management, targeting SpO2 more significant than 94%. Initiate O_2_ treatment at 5 L/min and titrated to reach the target or use a face mask with a reservoir bag (10–15 L/min) if the patients are in critical condition.

Once stable, the target is 90% SpO2 in non-pregnant adults and 95% in pregnant adults. The use of nasal prongs or nasal cannula is preferred in young children, as they may be better tolerated. When oxygen therapy fails, mechanical ventilation is necessary. In a meta-analysis, the use of additional oxygen therapy (38.9%), non-invasive (7.1%) and invasive ventilation (28.7%), and even ECMO (0.9%) was surprisingly high among the 1876 patients in which any kind of pharmacological and supportive intervention was reported [79].

#### Drugs

##### Antiviral agents

Remdesivir inhibits virus infection at the micromolar level (0.77–1.13 μM) and with high selectivity [80]. Remdesivir gets incorporated into viral RNA due to its adenosine analog nature and results in premature chain termination [81]. Remdesivir is not approved by the Food and Drug Administration (FDA). It is only recommended for mild or moderate COVID-19 conditions and the treatment of hospitalized adults and children in emergencies.

##### Chloroquine/hydroxychloroquine

Chloroquine increases endosomal pH, making the environment unfavorable for viral cell fusion. It also affects the glycosylation process of ACE-2. On administering chloroquine after 1 h of infection, gradual loss of antiviral activity was seen, though it affects the endosome fusion when administered shortly after the infection. When administered after 3–5 h after the infection, chloroquine was significantly effective against HCoV strain OC43 [82]. There is an excessive risk of toxicities due to high chloroquine doses; the recommended dose for chloroquine is 600 mg twice daily for 10 days for the treatment of COVID-19.

##### Interferon–alpha

Interferon-α is used in treating bronchiolitis; viral pneumonia; acute upper respiratory tract infection; hand, foot, and mouth disease; SARS; and other viral infections in children. According to the clinical research and experiences, the following usage is recommended for COVID-19

1. Interferon-α nebulization: interferon-α 200,000–400,000 IU/kg or 2–4 μg/kg in 2 mL sterile water, nebulization two times per day for 5–7 days
2. Interferon-α2b spray: applied for high-risk populations with close contact with suspected COVID-19 infected patients or those in the early phase with only upper respiratory tract symptoms.

##### Lopinavir/ritonavir

In a clinical trial among adult patients of or less than 18 years, it was observed that a combination of lopinavir/ritonavir, ribavirin, and interferon beta-1b would speed up the recovery, suppress the viral load, shorten hospitalization, and reduce mortality compared with lopinavir/ritonavir [83].

##### Immune-based therapy

Patients who show an inadequate response to initial therapy can get benefit from immunoglobulin [84]. Non-SARS-CoV-2-specific IVIG should not be used for COVID-19 except in case of clinical trials.

##### Corticosteroids

Corticosteroids are widely used in the symptomatic treatment of severe pneumonia. According to a detailed review and analysis, the result indicates that patients with severe conditions required corticosteroid therapy [85]. According to a systematic review of literature, daily use of corticosteroids in a COVID-19 patient is not encouraged; however, some studies suggest that methylprednisolone can reduce the mortality rate in more severe conditions, such as in ARDS [86].

##### Antimicrobial therapy

Patients with a mild type of bacterial infection can take oral antibiotics, such as cephalosporin or fluoroquinolones. Although a patient may be a suspect for COVID-19, appropriate antimicrobial agent should be administered within an hour of recognition of sepsis. Antibiotic therapy should be based on the clinical diagnosis of community-acquired pneumonia, healthcare-associated pneumonia, local epidemiology, susceptibility data, and national treatment guidelines. When there is the ongoing local circulation of seasonal influenza, this therapy with a neuraminidase inhibitor should be considered for the treatment for patients [87].

##### Tocilizumab

According to a review, 25 patients with laboratory-confirmed severe COVID-19 who received tocilizumab and completed 14 days of follow-up, 36% were discharged alive from the intensive care unit, and 12% died [88]. The biopsy specimen analysis suggested that increased alveolar exudates resulted from an immune response against an inflammatory cytokine storm. Probably an obstruction in alveolar gas exchange contributed to the high mortality rate of severe COVID-19 patients. A study identified that pathogenic T cells and inflammatory monocytes arouse an inflammatory storm with a large amount of interleukin 6. Tocilizumab blocks IL-6 receptors, which shows encouraging clinical results, including controlling temperature quickly and improved respiratory functions. Henceforth, tocilizumab is useful in the treatment of severe COVID-19 patients to calm the inflammatory storm and reduce mortality [89].

##### Ivermectin

FDA-approved drug ivermectin for parasitic infection has a possibility for reprocessing and acts as an inhibitor of SARS-CoV-2 in vitro. A single therapy can affect approximately 500-fold reduction and effectual loss of substantially all viral material by 48 h [90]. A single of ivermectin, in combination with doxycycline, yielded the near-miraculous result in curing the patients with COVID-19 virtually.

##### Azithromycin

Azithromycin is used for patients with viral pneumonia from COVID-19. It can also work synergistically and coactively with other antiviral treatments. It has also shown antiviral activity against the Zika virus and rhinoviruses, which cause the common cold. Viral infection was significantly reduced in patients receiving hydroxychloroquine than those who did not. The virus elimination was efficient in patients who received both azithromycin and hydroxychloroquine [91]. (Table 5) lists other supporting agents used in treatment [92].

**Table 5:** Supporting agents used in treatment

Antiviral agents	Supporting agents	Others
• Baloxavir • Chloroquine phosphate • Favipiravir • HIV protease inhibitors • Hydroxychloroquine • Neuraminidase inhibitor • Remdesivir • Umifenovir	• Anakinra • Azithromycin • Baricitinib (Olumiant®) • Colchicine • Corticosteroids (general) • COVID-19 • Convalescent plasma • Epoprostenol (inhaled) • Methylprednisolone (DEPO-Medrol®, SOLU-Medrol®) • Nitric oxide (inhaled) • Ruxolitinib (Jakafi®) • Sarilumab (Kefzara®) • Siltuximab (Sylvant®) • Sirolimus (Rapamune®) • Tocilizumab (Actemra®)	• ACE inhibitors, angiotensin II receptor blockers (ARBs) • Anticoagulants (low molecular weight heparin [LMWH], unfractionated heparin [UFH]) • Famotidine • HMG-CoA reductase inhibitors (statins) • Immune globulin (IGIV, IVIG, γ-globulin) • Ivermectin • Nebulized drugs • Niclosamide • Nitazoxanide • Nonsteroidal anti-inflammatory agents (NSAIDs) • Tissue plasminogen activator (t-PA; alteplase)

The repurposing of available therapeutic drugs is being used as supporting agents in the treatment of COVID-19; however, the efficacy of these treatments should be verified by using designed clinical trials

## Discussion

### Precautions and preventions

WHO declared the COVID-19 outbreak as a public health emergency of international concern on 30 January 2020. Unfortunately, no medication until now is approved by the FDA, and various trials are going on. Still, the most effective weapon the community has in hand is the prevention of spread. The following are some of the COVID-19 prevention measures.

- Quarantine: self-quarantine, mandatory quarantine (private residence, hospital, public institution, etc.)
- Other measures: avoiding crowding, hand hygiene, isolation, personal protective equipment, school/workplace measures/closures, social distancing [93].

Asymptomatic carriers as the “silent spreaders” are of great concern for the elimination of disease and its control. So, more attention should be given to them [94]. Hand hygiene with alcohol-based hand-rub is globally recommended as productive and economical procedures against SARS-CoV-2 cross-transmission [95]. The economic implications of hand hygiene have been established. It has been found that this cost under 1% of total HAI-related economics. It is better to invest not only in the materials needed but also in the people working there. This investment will lead to an increase in the health outcome [96]. The clinical presentation of COVID-19 is non-specific, so it needs a robust and accurate diagnosis. It has been suggested that before stopping the infection control measures, we have to be sure to exclude the diagnosis [97]. Prevention plays a vital role in treating and defeating the COVID-19 disaster.

**Fig. 8:**
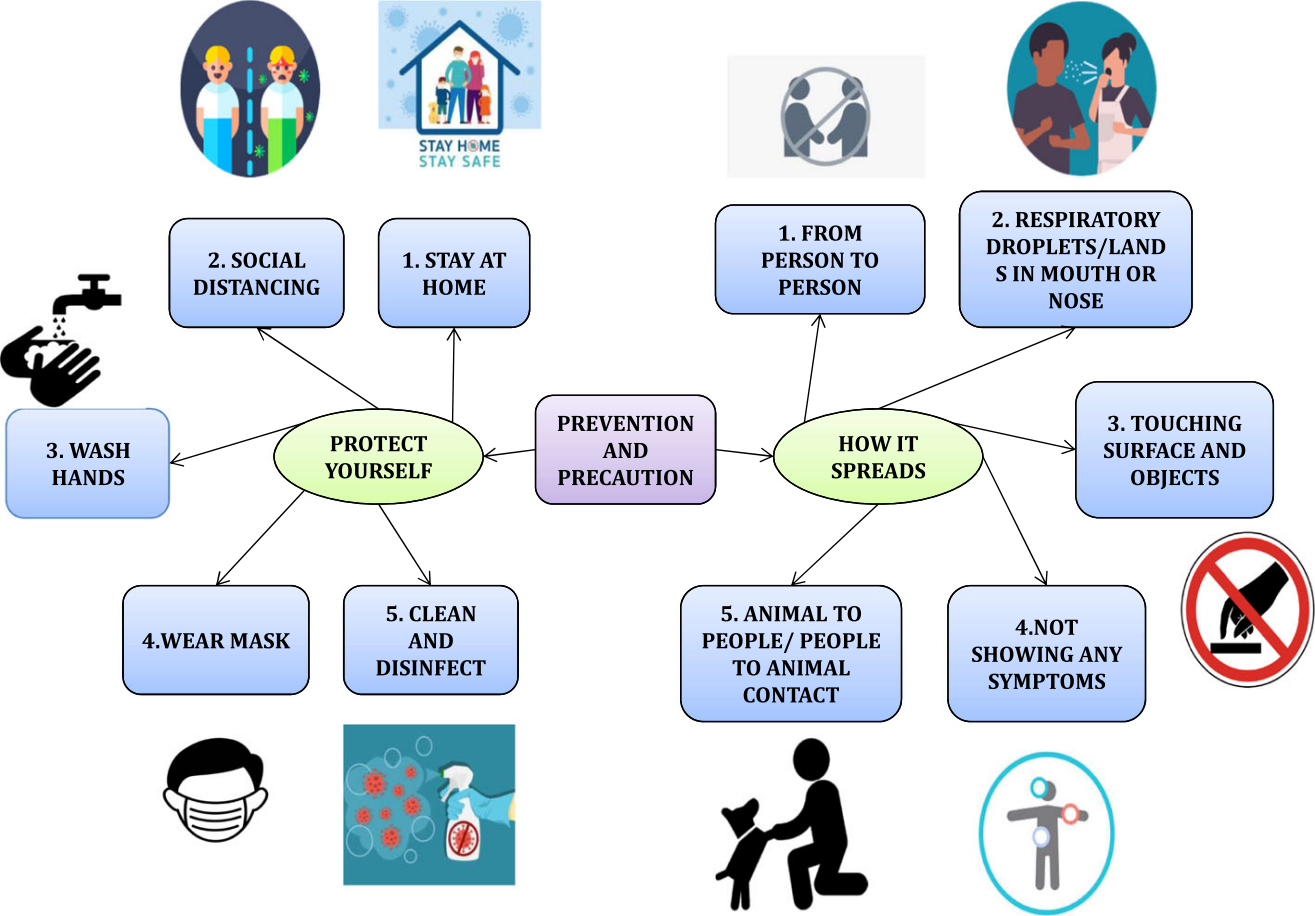
Prevention and precaution

The Centers for Disease Control and Prevention gives standard precautions (Fig. 8) and recommends measures to prevent COVID-19. Wear personnel protective equipment (face shield, mask, gown, gloves, and closed-toed shoes) when evaluating persons at risk. N-95 masks are known to prevent up to 95% of small particles, including viruses [98]. Cover all coughs/sneezes with a tissue and then throw the tissue away. Regularly clean/disinfect frequently touched objects and surfaces with household cleaning spray and use a tissue when handling (e.g., doorknobs, sink taps, water fountain handles, elevator buttons, cross-walk buttons, and shopping carts). Avoid contact with infected people (recommended > 6 ft) and maintain an appropriate distance as much as possible and refrain from touching nose eyes and mouth [99]. Avoid well persons when you are ill. Wear a mask continuously if taking care of persons with respiratory illness. To turn on the tap, use a paper towel and then wash hands with soap and water for at least 30 s after going to the bathroom. Use hand sanitizer and carry whenever at a public venue. Activate community-based interventions (e.g., cancel sporting events, dismiss, termination of universities and schools, practice social distancing, create employee plans to work remotely) [100]. Create a household-ready plan. Cancel any non-essential travel [101]. Frequent disinfection and cleaning are advised for groups that are at risk of contracting the virus [102].

In an Indian study mathematical approach was used to address some questions related to intervention strategies to control the COVID-19 transmission in India. Some hypothetical epidemic curves helped to illustrate the critical findings [103]. Predication of spread and implications of prevention and control using the Maximum-Hasting (MH) parameter assessment method and the modified Susceptible Exposed Infectious Recovered (SEIR) model was done. Suppression, mitigation, and mildness were the three predicted outlines for the spread of infection in some African countries [104].

Infection control strategies that can be acquired in hospitals were accomplished in a Taiwanese hospital to tackle the COVID-19 pandemic. These included emergency vigilance and responses from the hospital administration, education, surveillance, patient flow arrangement, the partition of hospital zones, and the prevention of a systemic shutdown by using the “divided cabin, divided flow” strategy. These measures may not be universally appropriate [105]. The preventive measures implemented in China included countrywide health education campaigns. The Examine and Approve Policy on the continuation of work, working and living quarters, a health Quick Response code system, community screening, and social distancing policies were some of the preventive measures [106].

Based on the analysis of immigration population data, the Epidemic Risk Time Series Model was outlined to estimate the effectiveness of COVID-19 epidemic control and prevention among different regions in China. Compared to other methods, this model was able to issue early recognition more instantaneously. For the prevention and control of COVID-19, this model can be generalized and applied to other countries [107]. The majority of clinical trials involving COVID-19 vaccines or treatment are showing encouraging results. (Tables 6 and 7) show ongoing phase 3 and 4 clinical trials [108].

**Table 6:** Ongoing clinical trials phase 3 studies

Study title	Conditions	Interventions	Locations
Randomized evaluation of COVID-19 therapy	Severe acute respiratory syndrome	Drugs: hydroxychloroquine, lopinavir/ritonavir, corticosteroid, azithromycin, tocilizumab	Nuffield Department of Population Health, University of Oxford, Oxford, UK
Hydroxychloroquine and zinc with either azithromycin or doxycycline for treatment of COVID-19 in outpatient setting *N*	COVID-19	Drugs: hydroxychloroquine, azithromycin, zinc sulfate, doxycycline	St. Francis Hospital, Roslyn, NY, USA
Favipiravir in hospitalized COVID-19 patients	COVID-19	Drugs: favipiravir, hydroxychloroquine	Shahid Modarres Hospital, Shahid Beheshti University of Medical Sciences and Health Services, Tehran, Iran
Baricitinib therapy in COVID-19	COVID-19 pneumonia	Drug: baricitinib 4 mg oral tablet	Fabrizio Cantini, Prato, Tuscany, Italy
Treatment for COVID-19 in high-risk adult outpatients	COVID-19 SARS-CoV-2	Drugs: ascorbic acid, hydroxychloroquine sulfate, azithromycin, folic acid	• Boston University, Boston, MA, USA • University of Washington Coordinating Center, Seattle, Washington, USA • UW Virology Research Clinic, Seattle, WA, USA and 4 more
Convalescent plasma for hospitalized adults with COVID-19 respiratory illness (CONCOR-1)	COVID-19	Other: convalescent plasma	• Vancouver General Hospital, Vancouver, British Columbia, Canada • Victoria General Hospital, Victoria, British Columbia, Canada • London Health Sciences Centre—University Hospital, London, Ontario, Canada and 25 more
BCG vaccine for health care workers as defense against COVID-19	Coronavirus infection, Coronavirus as the cause of diseases classified elsewhere	Biologicals: BCG vaccine, placebo vaccine	• Harvard T.H. Chan School of Public Health, Boston, MA, USA • Baylor College of Medicine, Houston, TX, USA • MD Anderson Cancer Center, Houston, TX, USA and 4 more
Outcomes related to COVID-19 treated with hydroxychloroquine among in-patients with symptomatic disease	Coronavirus acute respiratory infection -SARS-CoV infection	• Drugs: hydroxychloroquine, placebo	• Stanford University, Stanford, CA, USA • University of Colorado Hospital, Aurora, CO, USA • Denver Health Medical Center, Denver, CO, USA and 40 more
Treatment of COVID-19 patients with anti-interleukin drugs	COVID-19	• Other: usual care • Drugs: anakinra, siltuximab, tocilizumab	• University Hospital Saint-Pierre, Brussels, Belgium • University Hospital Antwerp, Edegem, Belgium • University Hospital Brussels, Jette, Belgium 13 more
Study to evaluate the safety and antiviral activity of remdesivir (GS-5734™) in participants with severe coronavirus disease (COVID-19)	COVID-19	Drug: remdesivir	• Kaiser Permanente Los Angeles Medical Center, 3340 E. La Palma Avenue, Anaheim, CA, USA • Alta Bates Summit Medical Center, Berkeley, CA, USA • Mills-Peninsula Medical Center, Burlingame, CA, USA and180 more

**Table 7:** Ongoing clinical trials, phase 4 studies

Study title	Conditions	Interventions	Locations
Evaluation of Ganovo (danoprevir) combined with ritonavir in the treatment of SARS-CoV-2 infection	COVID-19	Drug: Ganovo + ritonavir/interferon nebulization	• The Ninth Hospital of Nanchang, Nanchang, Jiangxi, China
The use of tocilizumab in the management of patients who have severe COVID-19 with suspected pulmonary hyper inflammation	COVID-19 pneumonia	Drug: tocilizumab	• Hadassah Medical Orginisation, Jerusalem, Israel • Barzilai Medical Center, Ashkelon, Israel • Wolfson Medical Center, Holon, Israel • Sheba Medical Center, Ramat Gan, Israel
Fluoxetine to reduce intubation and death after COVID19 infection	COVID-19 cytokine storm	Drug: fluoxetine	University of Toledo, Toledo, OH, USA
Hydroxychloroquine and zinc with either azithromycin or doxycycline for treatment of COVID-19 in outpatient setting	COVID-19	Drug: hydroxychloroquine, azithromycin, zinc sulfate, doxycycline	St Francis Hospital, Roslyn, NY, USA
Favipiravir in hospitalized COVID-19 patients	COVID-19	Drug: favipiravir, hydroxychloroquine	Shahid Modarres Hospital, Shahid Beheshti University of Medical Sciences and Health Services, Tehran, Iran
Azithromycin in hospitalized COVID-19 patients	COVID-19	Drug: hydroxychloroquine, azithromycin	Shahid Modarres Hospital, Shahid Beheshti University of Medical Sciences and Health Services, Tehran, Iran, Islamic Republic of
Prophylaxis of exposed COVID-19 individuals with mild symptoms using chloroquine compounds	• SARS-CoV2 • Symptomatic condition • COVID-19	• Drug: hydroxychloroquine sulfate regular dose, hydroxychloroquine sulfate loading dose, chloroquine, placebo	• Expo COVID Isolation Center/Mayo Hospital Field Hospital, Lahore, Punjab, Pakistan • Mayo Hospital/King Edward Medical University, Lahore, Punjab, Pakistan • Pakistan Kidney and Liver Institute, Lahore, Punjab, Pakistan
BCG vaccine for health care workers as defense against COVID 19	• Coronavirus • Coronavirus infection • Coronavirus as the cause of diseases classified elsewhere	• Biological: BCG vaccine • Biological: placebo vaccine	• Cedars-Sinai Medical Center, Los Angeles, CA, USA • Harvard T.H. Chan School of Public Health, Boston, MA, USA • Texas A&M Family Care Clinic, Bryan, TX, USA and 4 more
Hydroxychloroquine in patients with newly diagnosed COVID-19 compared to standard of care	• COVID-19 • Coronavirus Infection • SARS-CoV-2 • 2019-nCoV • 2019 novel coronavirus	• Drug: hydroxychloroquine • Dietary supplement: vitamin C	Portland Providence Medical Center, Portland, OR, USA
Efficacy of dexamethasone treatment for patients with ARDS caused by COVID-19	Acute respiratory distress syndrome caused by COVID-19	• Drug: dexamethasone	• ICU, Hospital Universitari Mutua Terrassa, Terrassa, Barcelona, Spain • Hospital Universitario Dr. Negrin, Las Palmas de Gran Canaria, Las Palmas, Spain • Department of Anesthesia, Hospital Universitario de Cruces, Barakaldo, Vizcaya, Spain and 21 more

### Impact of COVID-19 on overall health of the people worldwide

The international response to COVID-19 has been more transparent and efficient when compared to the SARS outbreak [109].

The pandemic COVID-19, being a most severe strainer, is affecting the overall health system worldwide. There is a continuously increasing demand for healthcare facilities and associated workers, which is overstretching the ability to operate efficiently [110]. Some pieces of evidence are showing a destructive effect on maternal and child health. Some financial, educational, sanitation, and even clinical constraints are threatening the overall population of the children [111]. As coronavirus is sweeping across the world, the primary psychological impact is elevated in terms of stress and anxiety. The quarantine period is expected to raise cases involving suicidal behavior, substance abuse, self-harm, depression, and loneliness. WHO Department of Mental Health and Substance use has given some messages to overcome psychological impacts [112]. There is a relationship between human development and infectious diseases. Whichever changes (new technology, constructions of dams, deforestations, migration, increasing populations, the emergence of urban ghettoes, globalization of food, and increasing international travel) brought about by the development, are stretching the word into the mouth of such pandemics indirectly. This pandemic is having a significant impact on the global economy as the erosion of capacity and rise in poverty [113, 114].

COVID-19 has affected the population differently based on gender. Significantly, this crisis is affecting the reproductive and sexual health of women. Another point is that there should be an equal contribution to both the genders in any healthcare body. There should be more distribution of decision-making power among them [115]. Protective measures can effectively prevent COVID-19 infection, including improving personal hygiene, wearing N95 masks, adequate rest, and proper ventilation [116].

### Have to learn to live with COVID-19

The Health Ministry has said that we have to learn to survive with COVID-19. We cannot step ahead by carrying the burden of COVID-19 that could recur annually and kill so many people [117]. Governments are learning to strike a balance between controlling COVID-19 spread and allowing individual freedoms and economic activity. Measures such as lockdowns, arbitrary travel bans, widespread quarantines, intrusive screening of people crossing boundaries can be adopted for prevention. Virtual work will become much more common. Supplier close-downs, sudden employee truancy, and demand collapse caused by disease outbreaks will make the businesses able to withstand disruptions.

The government, industry, or specialist certification for disease control processes and standards similar to ISO 9001 or USFDA certificate will be a crucial part of many businesses. The cost of traveling will expand more due to the risk of infection and lockdown. At the same time, the responsibility of work airlines, hotels, and restaurants will be added to minimize infection risk. Delivery businesses will perform well, and “Contactless delivery” is already a thing.

The industries that provide products to help circumvent, control, diminish, or treat COVID-19 will flourish. The requirement for hospital rooms will increase tremendously, with an increasing need for reserves of equipment, supplies, and drugs. In the upcoming time, businesses are likely to face demand crisis as the world comes to terms with living in a state of medical beleaguerment [118]. It is just a prediction, but we can still aspire for the best [119]. The most destructive effects would be in countries with weak health systems, on-going disputes, or existing infectious disease epidemics.

In contrast, the health systems in high-income countries would be stretched out by the outbreak [120]. It has been seen that resources are limited in countries with poor scientific infrastructure, such as Nepal, where there was only one laboratory equipped to test for coronavirus infection. Fear and stigma is an evident feature of the COVID-19; it has affected the economic and social development of many countries worldwide [121].

The insufficiency of the trained workforce capable of performing experiments required to test for SARS-CoV-2 and interpret the results is another major limitation in the testing and confinement of COVID-19 in developing countries [122]. The virus has the potential to adapt and get through the different environmental conditions, which makes it quite difficult to identify its mode of survival [123]. Another crucial impediment in a research project is a suitable model to investigate in vivo mechanism associated with the pathogenesis of SARS-CoV-2 [124–126].

Current screening approaches for COVID-19 are likely to miss approximately 50% of the infected cases, even in countries with sound health systems and available diagnostic capacities. Many symptoms correlated with COVID-19 are similar to malaria, such as fever, difficulty in breathing, fatigue, and headaches of acute onset. If symptoms alone are used to specify a case during the emergency period then, a malaria case may be misinterpreted as COVID-19. The symptoms for malaria are seen within 10–15 days after an infective bite; multi-organ failure is common in severe cases among adults, while respiratory distress is also expected in children [127].

## Conclusion

COVID-19 has emerged as the most terrified and enormous viral infection. According to WHO, the coronavirus might become an endemic disease. Originating from China as a global pandemic, it has influenced people on a large scale. There is no clear end that can be seen for this contagious disease. The only possible cure for this pandemic is prevention. We have to face it as a global community and support each other. The amplification of positivity will have a tremendous impact on the whole society. It is the duty of each individual for self-supervision and to report COVID-19 status, and challenging for those who appear to be ill. The other measure which can be followed to tackle this pandemic is healthy nourishment, sanitation, and hygiene practices robust connection and communication among children, and counseling to face the situation. Special care should be given to older people and pregnant ladies. It is better to get information only from the trusted sources; it is vital to get the facts and not the misinformation or rumors. Healthcare servants should have excellent and accurate communication with the public and must provide emotional and practical support. The ongoing pandemic of COVID-19 has caused not only notable morbidity and mortality in the world but also revealed significant systematic problems in the control and prevention of infectious diseases.

## Data Availability

The data and material are available upon request. The graphs and figures used in the manuscript were generated and analyzed and are not used anywhere else before.

## Abbreviations

ACE-2: Angiotensin-converting enzyme-2
ARDS: Acute respiratory distress syndrome
CoV: Coronavirus
COVID-19: Novel coronavirus infectious disease 2019
MERS-CoV: Middle East respiratory syndrome coronavirus
Nsps: non-structural proteins
ORF: Open reading frame
RBD: Receptor-binding domain
RTC: Replicase-transcriptase complex
SARS-CoV: Severe acute respiratory syndrome coronavirus
WHO: World Health Organization
